# Experiences of childhood weight management among Norwegian fathers of children with overweight or obesity – a qualitative interview study

**DOI:** 10.1080/17482631.2023.2235116

**Published:** 2023-07-13

**Authors:** Elin Salemonsen, Ingrid Oma Langeland, Anne Lise Holm

**Affiliations:** Faculty of Health and Social Sciences, Department of Health and Caring Science, Western Norway University of Applied Sciences, Haugesund, Norway

**Keywords:** childhood obesity, emotional barriers, father, shame, stigmatization, weight-management

## Abstract

**Objective:**

Paternal participation and experiences in childhood weight management is an understudied studied area. Given the important role fathers play in childhood obesity prevention and treatment, the aim of this study was to explore Norwegian fathers’ experiences of helping to prevent further weight gain in their children with overweight or obesity.

**Methods:**

Data were collected through semi-structured interviews with eight fathers of ten children with overweight or obesity and analysed by qualitative content analysis.

**Results:**

The analysis resulted in one overall theme: Balancing between assuming and avoiding responsibility for weight management with a desire to preserve the child’s dignity, comprising two themes: 1) Alternating between concern, helplessness and responsibility, 2) Needing acknowledgement, and flexible and tailored professional support, both of which have several sub-themes.

**Conclusion:**

Fathers need guidance on how to talk to their children to prevent further weight gain, while at the same time emphasizing safeguarding the child’s dignity. Healthcare professionals should address parents’ own emotional barriers and include fathers to a greater extent as a resource in family-centred counselling and tailor guidance and support to help with childhood weight management.

## 1 Introduction

The prevalence of overweight (ISO-BMI ≥25) or obesity (ISO-BMI ≥30) in children has nearly tripled since 1975. Worldwide, 39 million children under the age of five were afflicted by overweight or obesity in 2020. Over 340 million children and adolescents aged 5–19 were afflicted by overweight or obesity in 2016 (World Health Organization, 2021). In Norway, a total of 15–20% of children and 25% of adolescents are afflicted by overweight or obesity (The Norwegian Institute of Public Health, 2022). In low and middle-income countries, the BMI is still rising, and in many high-income countries, rising BMIs have plateaued, even if they are at high levels (Abarca-Gómez et al., 2017). Children with obesity tend to remain obese into adulthood (World Health Organization, 2016). According to the World Health Organization (WHO) (World Health Organization, 2021), obesity is preventable, however, psychosocial stressors and comorbidities may make behavioural change difficult (Gurnani et al., 2015).

Children with obesity are at a high risk of multiple comorbidities and childhood obesity is associated with a higher risk of poorer health, hypertension, insulin resistance, disability in adulthood and premature death (Kumar & Kelly, 2017; Sahoo et al., 2015; World Health Organization, 2021). Childhood obesity can affect children’s social and emotional wellbeing and self-esteem, and is associated with poor academic performance and lower quality of life (Sahoo et al., 2015; Steinsbekk, 2012). Weight-based bullying among young people is considered a common and serious problem in many countries (Puhl et al., 2016). Weight stigmatization may mediate negative health outcomes which in turn can be damaging to a child (Pont et al., 2017; Puhl & Latner, 2007; Sanyaolu et al., 2019). Many adolescents with obesity are socially marginalized (Strauss & Pollack, 2003) and experience bullying and fragile social relationships (Øen et al., 2018; Rankin et al., 2016). Adolescents with obesity report significantly higher bodily dissatisfaction, social isolation, symptoms of depression and negative self-esteem than people of a normal weight (Goldfield et al., 2010). Stigma contributes to behaviours such as social isolation, avoiding healthcare services, binge eating, decreased physical activity, and increased weight gain, which worsens obesity and creates additional barriers to healthy behaviour change (Pont et al., 2017). Some children who are overweight might seek emotional comfort in food, and stress in children is associated with emotional eating and more unhealthy dietary patterns (Michels et al., 2012).

Many children today are growing up in an obesogenic environment (World Health Organization, 2016). Increasing urbanization and technology developments have led to greater physical inactivity (World Health Organization, 2016), and energy-dense food, which is high in fat and sugars, is easily accessible. Obesity can be triggered by genetic, psychological, lifestyle, nutritional, hormonal and environmental factors. Environmental, genetic and societal factors have an impact on the development of overweight and obesity (Silventoinen et al., 2010; World Health Organization, 2016; World Health Organization, 2021). This also includes parenthood and family environment (East et al., 2019; Eg et al., 2017; Halliday et al., 2014; Mazzeschi et al., 2013; Sigman-Grant et al., 2015). Children’s eating and activity habits are influenced by their parents (Birch & Davison, 2001; Davison et al., 2018). Parents are responsible for their children, and for preventing overweight and obesity (Holm, 2008; Salemonsen et al., 2022; Wolfson et al., 2015). Research indicates that fathers play a key role in influencing children’s eating behaviour. Fathers’ dietary intake was a predictor of their children’s dietary intake, and fathers’ food-related parenting style predicted their children’s eating behaviour (Litchford et al., 2020). A previous interview study has found conflicting food-related parenting practices among 40% of the fathers involved in the study related to differences in parental eating habits, feeding philosophies and concern for the child’s health. These differences often resulted in tantrums or a refusal to eat (Khandpur et al., 2016). A literature review shows differences in mothers’ and fathers’ feeding practices. Fathers were generally less likely to monitor their children’s food intake and to limit access to food compared to mothers (Khandpur et al., 2014). Fathers are an important and valued part of a child’s life, and children whose fathers were highly involved in caregiving were less likely to be overweight (Sato et al., 2020).

Despite the expanding role of fathers in raising their children, they are underrepresented, compared to mothers, in child obesity research (Davison et al., 2018; Morgan et al., 2017). Qualitative empirical research concerning the role of the father in preventing obesity in his children is marginal, both in Norway and worldwide. Qualitative research methods examining fathers’ perceived roles and specific feeding strategies are required, and fathers should be routinely included in research on child feeding (Khandpur et al., 2014).

Knowledge about fathers’ experiences could be helpful for healthcare professionals (HPs) and public health nurses (PHNs) when they provide help and support to families and try to meet fathers’ needs in childhood weight management. Understanding perceptions and challenges of tackling childhood overweight and obesity among caregivers is critical to provide healthcare services in need. No studies have been found that describe Norwegian fathers’ weight management strategies or feeding practices, or how Norwegian fathers experience helping to prevent further weight gain, and what they need to help their children. Therefore, the aim of this study was to explore Norwegian fathers’ experiences of helping to prevent further weight gain in their children with overweight or obesity. The following research question was asked:

What do Norwegian fathers experience while trying to help and prevent further weight gain in their children?

## 2 Materials and method

### 2.1 Design

To increase understanding of fathers’ experiences of preventing further weight gain in their children with overweight or obesity, and how healthcare professionals may help, an explorative and interpretative design was chosen (Polit & Beck, 2021). Individual interviews were deliberately chosen to gain insight into experiences of fathers who know “where the problem lies … ”, and purposive sampling (Malterud et al., 2016) was used to find fathers with experience from their care of children with overweight or obesity.

### 2.2 Setting

Child health clinics and school health services in primary healthcare in Norway offer free advice and support from PHNs and general practitioners (GPs) to children and adolescents (aged 0–20 years) and their parents (Helsenorge, 2022). National guidelines recommend that GPs and PHNs in these child health services in primary care act at both the individual and structural level to prevent the development of overweight and help prevent and reduce obesity among children and adolescents (The Norwegian Directorate of Health, 2010). PHNs measure children’s height and weight at given consultations from birth to the age of 14 and are ready to follow up children and adolescents that have been identified as having overweight or obesity. Parents, children or adolescents are free to contact these free child health services if they need support or advice to manage the child’s weight.

### 2.3 Participants and recruitments

PHNs in 24 primary care child health services (local child health clinics or school health services) were asked to recruit fathers from families who had contacted them to obtain help for their children’s or adolescents’ weight, or from families where the family (either father, mother or both) were followed up by PHNs due to the child’s weight. The inclusion criteria were fathers of children who were afflicted by overweight or obesity (ISO-BMI >25), and who were involved in the family’s efforts to manage their child’s weight excess. The participants in this study were recruited from two school health services and three local child health clinics in three small (rural) and medium-sized (urban) municipalities. Our sample included eight fathers of ten overweight children. The children’s ages ranged from 4 to 16 years old. All the fathers have at least one overweight child, and all but one has daily custody of his child and live together with the child’s mother (Table I).

**Table I.** Participant characteristics.

Characteristics	Categories	Number of participants
Gender	Male	8
Age (34–55)	Mean age 45	8
Civil status	Single/divorced	1
	Partner/married	7
Education	Secondary school	6
	Bachelor’s degree or higher	2
Occupational status	Employee	8
	Daytime	4
	Shift work	4
Habitat	Rural	3
	Urban	5
Children/adolescents	Age	4–16

### 2.4 Data collection

Data were collected through individual interviews and the use of a semi-structured interview guide with follow-up questions. The form of the interviews was open, allowing for elaboration and follow up of the fathers’ statements. The interview guide was developed to explore how the fathers experienced taking care of their child or adolescent with overweight or obesity in order to prevent further weight gain, how they managed in everyday life and what they needed in their efforts to prevent further weight gain (Table II). The first author contacted all the fathers who had consented to participate after the PHNs had obtained informed and written consent for this purpose. All the interviews took place at the family’s local child health clinic based on the wishes and preferences of the fathers. After a short presentation, the purpose of the study was presented, and confidentiality was emphasized. The first author conducted eight individual interviews in Norwegian at three different local child health clinics. The interviews, which lasted between 80 and 90 minutes, were digitally recorded and transcribed verbatim by the first author. Information power, as discussed by Malterud et al (Malterud et al., 2016), such as the quality of the data, the narrow aim, access to participants and the nature of the topic, guided the sample size. The empirical data provided detailed and rich descriptions from the participants’ perspective.

**Table II.** Thematic guide for individual interviews.

What do fathers experience while trying to help and to prevent further weight gain in their children? How do you prefer to talk about your child’s overweight and with whom? What is your understanding of your child’s overweight? What do you perceive as difficult in childhood weight management? How do you talk to your child or adolescent about healthy diets, physical activity and weight? What do you need to help your child to prevent further weight gain? Have you been included in the local child health clinics? Who do you prefer to help you and your child?

### 2.5 Data analysis

Data were analysed by using qualitative content analysis (Graneheim & Lundman, 2004). The interviews were read as open-mindedly as possible to gain an overall understanding of the whole text, as well as an understanding of each interview. Several aspects of the fathers’ experiences of their efforts to prevent further weight gain in their children were identified. To handle the data in a systematic way as a coding process, a matrix was developed by the first author. The primary analysis, coding and categorization of the meaning units and preliminary themes were performed by the first author. Similar codes were grouped into categories and later sorted and abstracted into sub-themes. All authors discussed the identified codes, sub-themes and themes and made a tentative interpretation aiming to remain as close as possible to the meaning of the text. We discussed and agreed on interpreting the sub-themes and themes into one overall theme on a higher level of abstraction and interpretation (Graneheim et al., 2017; Lindgren et al., 2020). This abstraction can be seen as a presentation of the whole text. A number of quotations will be used to demonstrate the essence of the themes.

### 2.6 Trustworthiness and rigor

To ensure the trustworthiness of this study, the criteria credibility, dependability, confirmability and transferability, as put forward by Lincoln and Guba (Lincoln & Guba, 1985), were used. **Credibility** was strengthened through consistency in the research process, between the aim of the study, the participants, data collection and analysis. Direct quotations from the participants perspective demonstrated credibility in the interpretation of the data. **Dependability** deals with the stability and reliability of the data based on the potential for replication and was demonstrated through descriptions of the research process, including context, participants, data collection and analysis. **Confirmability** was strengthened by discussing the meaning-units, sub-themes and themes between the authors on several occasions to find the most appropriate interpretation. **Transferability** of this study’s findings to another setting or group of participants was demonstrated through descriptions of the study context and participants so that the reader can consider the relevance of the findings to other contexts.

### 2.7 Ethics

This study was approved by the Norwegian Centre for Research Data (NSD), project number 35008, and adheres to the requirements and ethical guidelines in the Helsinki Declaration. Informed consent was obtained from each father prior to the interviews. Participation in the study was voluntary and the participants were informed about their right to withdraw at any time, without this compromising their future healthcare.

We were aware of the potential reactions from the fathers when discussing this sensitive topic, as parents of children with excess weight are often blamed and shamed for their children’s weight (Gorlick et al., 2021). The first author, who conducted the interviews, was familiar with working with families and children afflicted by overweight or obesity. Before the individual interviews were held, precautions were taken by reflecting on how to take care of the participants if the interview situation became unpleasant or challenging. The interview setting was well-prepared beforehand, and emphasis was placed on creating a respectful, emphatic and non-judgemental atmosphere.

## 3 Results

The analysis yielded one overall theme: Balancing between assuming and avoiding responsibility for weight management with a desire to preserve the child’s dignity, comprising two themes: 1) *Alternating between concern, helplessness and responsibility*, 2) *Needing acknowledgement, and flexible and tailored professional support*, both of which have several sub-themes (Table III).

**Table III.** Overall theme, themes and sub-themes describing Norwegian fathers’ experiences of preventing further weight gain while caring for their child with overweight or obesity.

Overall theme: *Balancing between assuming and avoiding responsibility for weight management with a desire to preserve the child’s dignity*	
Sub-theme	Theme
Recognising their own food preferences and eating habits in their children	**Alternating between concern, helplessness and responsibility**
Struggling with their own feelings, shortcomings and discomfort	
Worrying about the consequences on psychosocial health	
A desire to help and protect	
Lack of arenas for conversation	**Needing acknowledgement, and flexible and tailored professional support**
A need for inclusion and acknowledgement	
Calling for flexibility and access to counselling and support	

### 3.1 Balancing between assuming and avoiding responsibility for weight management with a desire to preserve the child’s dignity

The analysis revealed that the fathers experienced substantial challenges in everyday life in their efforts to prevent further weight gain in their children. The fathers tried to balance between assuming and avoiding responsibility for weight management with a desire to preserve their children’s dignity. This overall theme is based on the fathers’ concern about their child’s psychosocial health, including self-esteem and stigma, being highly sensitive, and trying to avoid offending the child. They found it difficult to talk about the overweight and dietary restrictions with their child, fearing that they could inflict shame, guilt, or even contribute to their child’s eating disorder. The fathers felt that they were alternating between concern, helplessness and responsibility in an effort to prevent further weight gain, and that emotional barriers prevented them from adequate management. The fathers described a lack of arenas for conversation outside the family and expressed a need to be more included and acknowledged by health professionals. They called for a greater flexibility and access to counselling and professional support to manage their responsibility. These findings are further elaborated below. All quotations are assigned anonymously.

#### 3.1.1 Alternating between concern, helplessness and responsibility

The first theme described the fathers’ feelings of having shortcomings and helplessness due to their own emotional barriers, uncertainty and fear, and their concern for their child’s psychosocial health. They feared that they could inflict an extra burden on to the child or adolescent by addressing their weight gain or dietary restrictions. This helplessness and concern gave rise to an ambivalent feeling towards their perceived responsibility and how to help their children.

##### 3.1.1.1 Recognising their own food preferences and eating habits in their children

Most of the fathers recognized themselves in their children’s eating habits and some of them experienced having the same craving for sweets and soft drinks as their child did. Some of them described a sense of never being properly full. Several of them had experienced falling back on unhealthy habits and a feeling that overeating may be followed by shame and guilt. Some of them recognized themselves in their children’s search for something to eat in the cabinet, and some of the fathers admitted that they still do it themselves. One of the fathers said:

*… I slip … when the children have gone to bed … if there is an open pack of chocolate, it disappears… Or, when I come home from work, hungry and making dinner … I hunt for something sweet to eat myself … I try not to show the kids; I eat when they are not present … (P5)*

Another father said:

*… he has some bad habits that I can see in myself … when we go to parties and food and cakes are served as a buffet … both my son and I have this tendency to eat alot … this is my son’s weak point, and this is my weak point [as well]. I almost never get enough! (P3)*

##### 3.1.1.2 Struggling with their own feelings, shortcomings and discomfort

Several of the fathers described that having an overweight child and having experienced being overweight themselves often generated negative feelings. Some of them said that they found it unfair that they put on weight more easily, and that they always needed to be careful with what they ate or needed to be “on their guard”. Several of the fathers described how bad they felt if they had to limit the amount of food or say no to their children. This gave them a bad conscience and made them feel uncomfortable.

*I find it difficult … when you know there is something they like very much, and they ask for more, and you always have to say no … that is not easy …. (P7)*

Some of the fathers described soreness and pain, both in themselves and in their children. These feelings lead to ambivalence and emotional barriers, which prevent them from adequate coping. One of the fathers stated:

*… She asks us if we think that she is fat, and I tell her that she is great. I think she is likely to accept this, but I don’t know. It hurts me a bit when she asks about it, it is not that nice that they have this problem … but we often send contradictory signals: “I say you are fine, but you are not allowed to eat” …. (P8)*

Other fathers described irritation, anger and frustration about their children not being able to restrain themselves. One of them said:

*… My son cannot restrain himself when it comes to sweets and soda, and he is addicted to sugar. I might feel annoyed when he fails to restrain himself, but I recognize it in myself. I want to say to him: … “you have to understand that you can’t sit and eat that much! You have to control yourself!” (P3)*

Several of the fathers found it important that there should be a pleasant atmosphere around the dinner table. Several of them said that they are permissive just to keep a peaceful atmosphere when they eat. Many of the fathers expressed that mothers were most strict and most consistent, both in relation to the food that was purchased and offered in the family. One of the fathers said:

*… Mothers are maybe stricter than fathers … but we try to do the same at our home, but maybe it’s me giving in … Can’t they just get some more so we can have a peaceful meal … instead of arguing at the table. (P7)*

The fathers found it difficult to talk about the weight and dietary restrictions with their child, fearing that they could inflict shame or guilt and even contribute to their child’s eating disorder. They were able to talk to their partner; however, when it came to their child, they started to feel uncertain and afraid. The uncertainty was related to figuring out the right way to say it and finding the right words with which to approach the topic with their children. The fear was related to them potentially expressing themselves in the wrong way and focusing too much on the overweight, and the fact that they could inflict an extra burden, and in the worst case scenario, psychosocial issues on their children. One of the fathers said:

*… there has been this fear, that we have to be careful … that we have to find the right words … so that he doesn’t end up with an eating disorder … (P3)*

##### 3.1.1.3 Worrying about the consequences on psychosocial health

All the father’s described numerous concerns about the consequences of overweight and obesity, whether it be physical, social and mental health risks. Regarding physical health, they were concerned that cardiovascular diseases and diabetes could occur later in life. The fathers had experienced consequences for their children’s participation in sports and performance in different activities and described that their children did not manage to run as much or as fast as other children and that they are left behind. However, at this point, it was primarily the social and psychological consequences for their children’s health that worried them most. All the fathers described a deep concern about their children’s self-esteem, stigmatization and psychosocial health. One of the fathers said:

*… I see that she is very sensitive to this. If her sister is getting a little rude, and I know* *if they start a quarrel, she will call her “fat girl” … and I see that it hurts, you know … (P8)*

Two of the fathers described that they had struggled with overweight themselves since childhood. It was their sincere desire that their own children should avoid the same experiences as them and take the overweight with them into adulthood. One of the fathers said that his son, like himself, never found trousers that fitted, which damaged his body image and self-esteem. According to the fathers, most of the children had already experienced teasing, bullying and stigmatization, and they had observed that this affected their children in different ways and to different degrees. One of the fathers stated:

*I believe that he has got some comments at school, and I am sure that he is aware of his big body. He does not run as fast as his mates, and he does not jump as high as his mates. This impacts his body-image and self-esteem … I have personally experienced being fat and I wish that my son could avoid this. (P3)*

##### 3.1.1.4 A desire to help and protect

The fathers expressed a concern that the overweight will be a lasting challenge and wanted to help their children so that they could avoid having to struggle with this for the rest of their lives. Several of them find it important to be open and that more people should get to know what other people struggled with. Some of the fathers choose to focus on a healthy diet rather than the child’s weight and choose not to talk about their children’s weight with the child. The fathers acknowledged their responsibility as parents on equal footing as mothers, and that they felt responsible for having and maintaining a healthy lifestyle within the family. Several of the fathers found it important to take their time to plan, to have regular mealtimes and to be structured in their everyday lives. They believed this would help them to create healthy habits for their children and family early on, and it could help them to eat less fast-food and prevent impulse buying. All the fathers underlined the importance of not having sweets and high-density food such as cookies, chocolate or potato crisps available at home. However, several of the fathers found it challenging to be a good role model, always planning healthy meals and engaging in activities and sports. One of the fathers stated:

*We try to make a good plan for the meals in our family, which includes a healthy diet and sticking to the shopping list … (P5)*

All the fathers highlighted the importance of giving the children’s confidence and belief in themselves through respect, encouragement and positive involvement in their children’s life. Most of them underlined the importance of being good role-models. One of the fathers stated:

*I have this urge to connect with my kids. It is important to be part of their activities, yes, because it provides confidence. I see that the more confident they are in themselves, the more responsibility they take for their own life… yes, it is important that they believe in themselves. (P1)*

#### 3.1.2 Needing acknowledgement, and flexible and tailored professional support

The second theme described the fathers’ perceived lack of arenas for conversation, their need for acknowledgement for their perceived parental role, and a desire to be included in consultations. In addition, they asked for flexible and tailored professional support in their efforts to prevent further weight gain in their children.

##### 3.1.2.1 Lack of arenas for conversation

Most of the fathers find it perfectly natural to talk about their children’s diet, activity and overweight with the child’s mother. Several of the fathers felt, however, that it is not natural to talk to other fathers about their children’s diet or problems. Several of them described that they were most comfortable discussing activities, sports and handcrafts with other fathers. They believed that mothers have more natural arenas and networks to talk about this issue than fathers have.

*… as a father I don’t use those channels as she does. She talks much more with other mothers about weight, diet and … fathers don’t when they come together. We don’t talk about sensible things like that. We talk about football and sports … not bread toppings and weight …(P4).*

##### 3.1.2.2 A need for inclusion and acknowledgement

The fathers in this study described that they are more engaged in their children’s life than their parents were. They described keeping up with their children at home, at school, with homework and in leisure activities, and believed that they have an equally important parental role as mothers do. Some of the fathers expressed a need for greater acknowledgement of their parenting role in general, and all the fathers wanted to be included and involved in the family’s efforts to prevent and treat their children’s overweight or obesity. However, they experienced that all information and communication between the child health services and the family went through the child’s mother. Some of them described a feeling of being set aside, and one of the fathers felt that there was a certain degree of discrimination since the kindergarten, school and the child health services always contacted his child’s mother first. Most of the fathers wanted to be more included by health professionals and to receive guidance on discussing this problem with their children, without imposing additional burdens on their child:

*I believe that our public health nurse at the child health clinic could help us to manage our daughter’s weight problem and give us some advice on how to talk to her. “What is the best way to deal with this problem?” … however, they could be better at contacting and including me, not only the child’s mother …. (P6)*

##### 3.1.2.3 Calling for flexibility and access to counselling and support

Several of the fathers had experienced that it could be difficult to attend counselling at the child health clinics due to their work. Most of them expressed an understanding of the difficulties in organizing extended opening hours, however, they hoped for greater flexibility. Some of the fathers requested counselling in the afternoon and believed that it would be easier for them to attend appointments after regular working hours.

“*It is quite difficult for me to attend to a meeting or a counselling session at the child health clinic during working hours. It would be much easier for me to attend in the afternoon”. (P3)*

## 4 Discussion

In this study we asked Norwegian fathers what they experienced while trying to help and to prevent further weight gain in their children with overweight or obesity. In this section, we will discuss the results, and, in the light of these experiences, discuss how HPs may help fathers to prevent further weight gain in their children. The overall theme and the two themes will guide the discussion.

The overall theme *Balancing between assuming and avoiding responsibility for weight management with a desire to preserve the child’s dignity* reflects what the fathers in this study experienced when they tried to prevent further weight gain in their children. The fathers expressed a deep concern about their child’s self-esteem and psychosocial health. They were highly sensitive towards their child’s weight challenges and tried not to offend their child. The results suggest that, in essence, the fathers want their children to have dignity so that they can improve their self-image and maintain their integrity. Dignity can be related to self-esteem, as it refers to the worth of human beings, and the right to be valued and respected. Dignity is the opinion of others about our worth, and dignity can be understood as our subjective fear of the opinions of others (Schopenhauer & Saunders, 2004). For all parents, their child’s worth is important. To preserve the child’s dignity, we suggest that the fathers in this study tried to create a balance between assuming and avoiding responsibility. This balancing act can be understood as an effort to manage the emotional burden of experiencing their children’s challenges, like stigma and low self-esteem. In addition, some of the fathers recognized themselves in their children’s issues, including their own previous and current feelings of guilt, shame or weight stigma. Parents may feel blamed or shamed for their children’s weight (Wolfson et al., 2015). A previous study shows that mothers can be the target of weight stigma and can experience negative emotions like blame or shame due to their perceived role and responsibility regarding their children’s weight (Gorlick et al., 2021). Based on the father’s descriptions of their concern, helplessness, perceived responsibility and emotional distress, we suggest that this blame or guilt applies equally to fathers and may lead to an avoidance of responsibility. Avoidance of responsibility may also be seen as an emotional defence to their distress. Management and pride in an active involvement in their children’s issues, as well as their wellbeing, may help the fathers to assume responsibility. Several of the fathers highlighted the importance of giving their children belief in themselves and increasing the children’s self-efficacy, which is something they try to do. Both shame and self-efficacy are constructs closely tied to the foundation of the self (Lewis, 1971). Reducing feelings of shame or increasing self-efficacy is a two-sided process, and shame and self-efficacy are correlated (Baldwin et al., 2006). A new direction of treating shame “through the backdoor” is to improve self-efficacy (or the treatment of either aspect), and this could positively impact the other aspect. By helping people heal from shame, self-efficacy could be raised, and by helping to raise self-efficacy, shame could be reduced (Baldwin et al., 2006). To help fathers help their children and to prevent further weight gain, HPs need to address self-conscious feelings like shame, internalized stigma, as well as the feeling of responsibility related to dilemmas about how to talk about food restrictions with their children, for example.

Several of the fathers found it difficult to talk about overweight and dietary restrictions with their child, fearing that they could inflict feelings of shame or guilt on them, or even contribute to their child’s eating disorder. As our results revealed, the fathers were uncomfortable talking to their children about food restrictions and avoided this situation. Several of them said that they are permissive just to keep a peaceful atmosphere when they eat. This is in line with the previously mentioned literature review that showed differences in mothers and fathers feeding practices, where fathers were generally less likely to monitor children’s food intake and to limit access to food compared to mothers (Khandpur et al., 2014). The fathers in our study have helped to contribute to deepening our understanding of this complex and difficult parental responsibility, the ambivalence they feel and the strong desire to preserve the child’s dignity and to have a peaceful and harmonious everyday life for their family. We know that conflicts may generate more problems like tantrums or a refusal to eat (Khandpur et al., 2016). Previous studies (Eg et al., 2017; Salemonsen et al., 2022) show that fathers believed that agreement between parents and minimizing conflicts are important for managing lifestyle changes, and that the family climate affected the child’s eating habits. Dysfunction in parental alliances and family functions could be a predictor of overweight and obesity in adolescence (Mazzeschi et al., 2013). These findings are in line with the perceptions and experiences of the fathers on the importance of support, collaboration and alliance between the parents (Salemonsen et al., 2022). The fathers in our study seemed to intuitively understand this association, believing that it would not benefit the child to have high conflicts and a warzone-like atmosphere at mealtimes, and they are permissive in order to protect the child’s dignity. At the same time, this fear of conflict and almost an avoidance of responsibility could be a strong call for help to receive guidance on how to deal with food restrictions and how to approach this delicate and difficult challenge. In addition, this reflects the complexity in childhood weight management and shows how emotional barriers and ambivalence may lead to the alternation between concern, helplessness and assuming and avoiding responsibility.

The second theme *Needing acknowledgement, and flexible and tailored professional support* reflects the fathers’ need for help and guidance in their caregiving and efforts to prevent further weight gain in their children. Our findings illustrate that the fathers want to be involved, to help, contribute to a better life and protect their children. They wanted to be included and to receive guidance on discussing this problem with their children, without imposing an additional burden on their child. The PHNs and child health services are expected to follow up parents in primary care (The Norwegian Directorate of Health, 2017). Despite fathers’ wishes to be included and their perception of their equal parental role and responsibility, this study reveals that the fathers do not have the same access to support at child health clinics. They are not included in the same way as mothers since they do not receive information and other correspondence from the child health services. Since the fathers described a lack of arenas for conversation outside the family and expressed a need to be more included and acknowledged by HPs, it will be necessary to facilitate greater flexibility and access to counselling and professional support to help them manage their responsibility. A previous study (Lowenstein et al., 2013) explored fathers’ perceptions and experiences of communicating with their children’s healthcare provider during visits to clinics regarding weight, diet and physical activity. They found that fathers were involved in their children’s healthcare and that fathers found providers to be helpful partners, depending on the quality of this relationship. However, they felt “left out” during clinic appointments. Other studies found that fathers want to play a more active role during pregnancy, childbirth and follow-up at the child health clinic, however they felt excluded by HPs (Høgmo et al., 2021; Solberg & Glavin, 2018). This requires a change in child healthcare clinics and services and that they treat fathers as independent and equal carers, and provisions should be made for fathers to be included to a greater degree. To help involve and include fathers, HPs can provide them with a specific invitation to an appointment (Ahmann, 2006). Work conflicts for fathers and greater convenience for mothers accompanying children to healthcare visits is described (Ahmann, 2006). Flexibility in counselling may be a way to meet fathers’ needs by, for example, trying to find a time at the beginning of the day or at the end of the clinic’s opening hours, and by inviting the fathers to the clinic personally, and not just sending a message through mothers. HPs may increase the father’s self-efficacy by facilitating support, both emotional and practical support. This may be an important therapeutic contribution to reduce blame or shame in parents that is related to their child’s weight excess.

Reducing the feeling of shame or blame may help parents assume responsibility. Most of the fathers in our study emphasized focusing on a healthy lifestyle, including a healthy diet and physical activity, and not putting so much focus on weight. According to Golden et al (Golden et al., 2016), guidance about messages around obesity and eating disorders should focus on a healthy lifestyle rather than on weight. Evidence suggests that obesity prevention and treatment, if done correctly, will not lead to someone developing an eating disorder (Golden et al., 2016). Questions from the fathers regarding the “right” or “correct” way to do this are appropriate questions to ask, as there is a general and established concern from both parents and health professionals regarding the uncertainty of what factors can contribute to eating disorders. One way of helping these families, especially the fathers, is for HPs to address the fathers’ own feelings of guilt, shame and also their concerns, feelings of helplessness and discomfort. This could be done by boosting the fathers sense of self-worth through long-term support from competent and sensitive HPs. The support must be based on dialogue and a non-judgemental attitude. Studies exploring beneficial support in weight-management show that dialogue, a non-judgemental attitude and a shared responsibility is useful for self-management (Salemonsen et al., 2020). Using nonbiased language, empathic and empowering counselling techniques and addressing stigma and bullying in the clinic visits are also recommended (Pont et al., 2017). To facilitate weight management, continued support from HPs should be offered to parents (Nowicka et al., 2022). Inclusion, acknowledgement and support from HPs tailored to the fathers and the families’ needs, may help the fathers in preventing further weight gain in their children. Tailored and professional support may focus on the recommendations from Neumark-Sztainer (Neumark-Sztainer, 2009), which emphasize promoting a positive body-image and encouraging more enjoyable meals. Puhl et al. (Puhl et al., 2023) found that most parents wanted guidance on how to navigate weight related topics, including promoting healthy behaviours and positive body-image, and that there is a need for education to help parents engage in supportive conversations about body weight.

## 5 Strengths and limitations

No studies have been found that explore Norwegian fathers’ feeding practices or how Norwegian fathers experience helping prevent further weight gain in their children, and what they need in order to help their children. Our study is the first study to explore Norwegian fathers’ experiences in weight management for their children, and adds to the field of parental experiences, especially fathers’ experiences, of caring for their child in order to prevent further weight gain. The strength of the study is that it provides knowledge exclusively from the fathers’ perspectives and gives a deeper understanding of the emotional challenges that influence weight management. This knowledge about fathers’ experiences in childhood weight management could be helpful for HPs and PHNs when they provide help and support to families; to meet the fathers’ needs in overweight and obesity management and to increase HPs own awareness of paternal concerns and needs. This insight into the fathers’ own emotional barriers may help HPs to provide tailored guidance and support and to better understand the necessity of acknowledging, involving and including fathers. This paper meets the requirements of the COREQ checklist (Tong et al., 2007).

However, some limitations should be addressed. Fathers self-selected to participate in our study and may have been more engaged and involved in caring for their children than other fathers with the same challenges. Several of the fathers had participated in group or individual consultations with a PHN, GP or paediatrician over a period of time prior to the interviews, and they exhibited a high degree of self-reflection and knowledge. This may reflect a selection bias and hence limit the transferability of the findings (Lincoln & Guba, 1985). The fathers have not been actively involved throughout the research process.

## 6 Conclusion and implications for clinical practice

The fathers in this study wanted to care for and help their children with overweight or obesity to prevent further weight gain. However, they found it difficult to talk about obesity and dietary restrictions with their child, fearing that they could inflict shame or guilt on them and even contribute to their child’s eating disorder. The fathers expressed deep concerns about their children’s self-esteem and psychosocial health. They felt that they were alternating between concern, helplessness and responsibility, and that emotional barriers, ambivalence and concerns prevented them from providing adequate weight management. The fathers tried to find a balance between assuming and avoiding responsibility for weight management with a desire to preserve the child’s dignity.

In order to help fathers prevent further weight gain in their children, fathers need guidance on how to talk to their children about food restrictions, for example, while at the same time emphasizing safeguarding their children’s dignity, and without imposing additional burdens on the child. The fathers described a lack of arenas for conversation outside of the family and expressed a need to be included and acknowledged by HPs and have access to counselling and professional support. HPs should address parents’ own emotional barriers and include fathers to a greater degree as a resource in family-centred counselling to help prevent and treat childhood obesity. To involve the fathers in counselling related to childhood overweight and obesity, HPs may need to provide tailored long-term emotional support, in addition to practical support and useful tools on how parents can communicate with their children on this topic. This will require competent and sensitive HPs who base their support on dialogue, a non-judgemental attitude and best practice.

## Data Availability

The dataset used and analysed during the current study is available from the corresponding author on reasonable request due to ethical restrictions.

## Abbreviations

GP: General Practitioners
HP: Healthcare professionals
PHN: Public health nurse
